# Editorial: Mathematical, Computational, and Empirical Approaches to Exploring Neuronal Mechanisms Underlying Cognitive Functions

**DOI:** 10.3389/fnhum.2022.896213

**Published:** 2022-05-03

**Authors:** Vipin Srivastava, David J. Parker

**Affiliations:** ^1^School of Physics, University of Hyderabad, Hyderabad, India; ^2^Department of Physiology, Development and Neuroscience, University of Cambridge, Cambridge, United Kingdom

**Keywords:** empirical approach, mathematical models, computational models, cognitive function, neuroscience

The idea behind doing a special issue of Frontiers in Human Neuroscience on the above Research Topic was to promote the empirical approach in cognitive neuroscience wherein a mathematical or a computational model is formulated for a given cognitive process, quantitative results are derived and compared with experimental results where they are available, or suitable experiments are proposed to test assumptions. The scope for works that can be covered by this approach in the domain of cognitive neuroscience is endless because of the interdisciplinary nature of the subject.

Mathematical and computational modeling allow great flexibility in which one can resort to simplifications and approximations to make a model tractable and get a preliminary idea of what underlies a cognitive phenomenon that is to be explored. The model is then brought closer to being more and more realistic by relaxing the approximations and simplifications systematically. At every stage, either intuitively or through experiments, the explanations given are tested against the actual system. In the limit in which a formal apparatus explains almost all the conceivable facets of the cognitive phenomenon being studied, it qualifies to be called a “theory”.

The combination of formal and experimental methods has a long history in neuroscience. The interdisciplinary nature of cognitive neuroscience makes it ideal for the iterative application of mathematical and experimental approaches. This was the motivation for this venture.

The initial response to call for papers was very good. However, the enthusiasm soon began to fade as the prospective contributors expressed their inability to pay the publication charges. The venture was rescued by the intervention of the top administration of the journal but not before most of the authors had withdrawn and could not be persuaded to join in spite of the fee waiver. A couple of papers were rejected in the peer reviewing process. Eventually, we had to content with five papers, but all are of very high standard and represent an area of research in their own right.

## Summary in a Nutshell

Philosophical inquiry is crucial to any scientific endeavor, more so to the understanding of the abstract cognitive phenomena. Philosopher Amitabha (Das Gupta) of University of Hyderabad gives a new dimension to our understanding of cognition by our mind by arguing that cognition has a multifaceted character and thus it cannot be restricted to any one narrow perspective, such as the prevailing disembodied or mentalistic approach based on mechanical concept of mind. He pleads that cognition is embodied in that the body has a fundamental epistemological function since our cognitive access to the world is mediated by body. Besides the philosophical part that human cognition cannot be dissociated from its bodily basis, he brings in the empirical part to confirm the conceptual claims of the first part of the inquiry.

Taking the philosophical inquiry further quantum chemist (Brändas) from Uppsala attempts to link the brain and the mind by extending the platform of quantum mechanics to open state dynamics using non-equilibrium quantum statistical mechanics. He invokes esoteric Gödel's incompleteness theorem and quantum Darwinism to deal with model dependent axioms.

In a down to earth approach the Oxford Cognitive Psycholinguists (Duta and Plunkett) present a neural network model that processes words incrementally and associates them with internal lexical, semantic, and visual representations. Their hypothesis is that incremental unfolding of a spoken word is in itself sufficient to account for the transient preference for phonological competitors over both unrelated and semantically and visually related ones.

Continuing in the same spirit, cognitive neuroscientist, Supriya Ray from University of Allahabad and his student Pragya Pandey wonder if the pupil size is controlled only by light or whether it is also affected by the state of our mind (Pandey and Ray). They use both computational and empirical approaches to study how covert orientation of attention, without shifting gaze, to a visual stimulus influences changes in the pupil size. They go further and study the mechanism by developing a homoeomorphic bio-mechanical model of pupillary muscle plants.

We would like to end by summarizing a curious study reported by computational neuroscientist S Bapi Raju of International Institute of Information Technology at Hyderabad and his collaborators about the much speculated effect of meditation on dementia, which may be caused by mild cognitive impairments (MCI) or early Alzheimer's disease (AD) (Dwivedi et al.). They use a statistical measure called symmetrized percentage change (SPC) on the longitudinally (6 months) acquired structural imaging data from two groups of MCI/AD patients—meditators and non-meditators. SPC allows for comparisons even with variable intervals of follow-up data acquisition. Their results indicate improvements in the meditation group in the structural features (cortical thickness and gray matter volume) of the brain areas related to executive control and memory. The results assume significance in view of the fact that dementia currently has no cure to stop or even delay its progression.

## Author Contributions

All authors listed have made a substantial, direct, and intellectual contribution to the work and approved it for publication.

## Conflict of Interest

The authors declare that the research was conducted in the absence of any commercial or financial relationships that could be construed as a potential conflict of interest.

## Publisher's Note

All claims expressed in this article are solely those of the authors and do not necessarily represent those of their affiliated organizations, or those of the publisher, the editors and the reviewers. Any product that may be evaluated in this article, or claim that may be made by its manufacturer, is not guaranteed or endorsed by the publisher.

